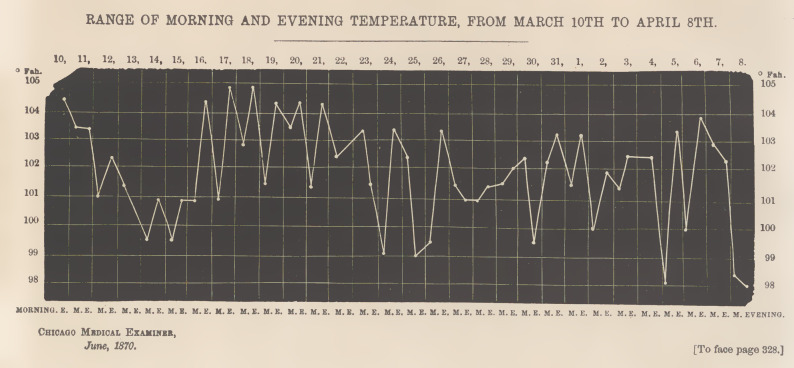# Typhoid Fever, Its Varieties and Treatment

**Published:** 1870-06

**Authors:** A. Given

**Affiliations:** Louisville, Kentucky


### CHICAGO MEDICAL EXAMINER.

N. S. DAVIS, M.D., Editor.

VOL. XI. JUNE, 1870. NO. 6.

### Original Contributions.

ARTICLE XVI.

### TYPHOID FEVER, ITS VARIETIES AND TREATMENT.

By A. GIVEN, M.D., Louisville, Kentucky.

This is an idiopathic disease, which is characterized by a forming stage, in which the zymotic agent is at work in the system, from one to ten days before the fever is fully developed. After the stage of febrile excitement has been established, the fever is continuous, and the disease runs its course with a diversity of symptoms that are pathognomonic of typhoid fever and its complications.

This disease has long been known, but was imperfectly understood, until after the research of Louis, who established the fact of its constant anatomical and pathological pecularity. The writers who preceded him discribed it as a variety of typhus, and many of the European authors still so regard it. But their individuality or non-identity is settled beyond the shadow of a doubt.

The nomenclature of this disease is vague. Writers have diligently sought for a name expressive of the pathological condition, but, as yet, none has been found free from objection. Probably the one that is least objectionable is "enteric;" but this does not convey a definite idea of the character of the disease under consideration.

The term typhoid has become so popular, both among the people and profession, that it will be somewhat difficult to change it. But the distinction must ever be kept in view, between this fever and that secondary condition of many diseases, called the typhoid state.

Typhoid fever is primarily a disease of the blood—that is to say, a zymotic action is set up in that fluid, by the presence of the typhoid ferment, by which the fever is developed.

It would be natural to suppose that all the tissues of the body would be more or less affected by a disease so general in its character. But it seems to have an affinity for certain localities, through which the vital powers are more readily reached, and so depressed as frequently to cause the death of the patient.

In whatever country typhoid fever prevails, or whatever the character of the epidemic, it has phenomena peculiarly its own. But each epidemic may differ in its local manifestations. At one time, the disease may expend its force upon the organic functions collectively, while, in other cases, it may select a particular organ for its base of operations.

The abdominal, cerebral, gastric, and respiratory apparatus are the parts most generally affected by typhoid fever. I will, therefore, notice six varieties or conditions, which demand special consideration. The first stage or premonitory symptoms are similar in all; the complication or variety is soon developed, and gives to the disease its peculiar characteristics. The predisposing cause so depresses the vital affinity, and increases the susceptibility of the fluids and solids, that the morbific agent, in passing the rounds of the circulation, selects the weakest part, and there concentrates its forces.

For convenience of description, I make the following tabular view of this fever and its subdivisions:—

| | |
|---|---|
| TYPHOID FEVER, ------ | Simple Typhoid,<br>Typho-enteric,<br>Typho-cerebral,<br>Typho-gastric,<br>Typho-pneumonitis,<br>Typho-malarial. |

### SIMPLE TYPHOID.

Under this head, I propose to notice the clinical history and symptoms that belong to typhoid fever, independently of the complications to which I have referred.

The approach of this disease is so gradual, that we are often unable to tell the precise time of its beginning. The patient complains of a general indisposition and lassitude; there is an aversion to exercise, both mental and physical; the appetite is impaired; the bowels are generally loose; there may be but one stool during the 24 hours, and that is thin and watery. If the bowels do not move, the patient complains of borborygmus; and a small amount of physic will produce the thin and watery stools so characteristic of this disease. There is a little tenderness in the right iliac region, which may be overlooked, in the early stage, unless a close examination be made by pressure. It is often so mild that the patient does not complain, and is not aware of any trouble in that region, until his attention is called to the fact by the physician. Headache is often present, but this seldom in the early stage; the tongue is covered with whitish fur; the pulse is only a few beats above the normal standard; slight pains are felt in the back and limbs. The patient often continues about his employment, in this condition, from one to ten days, when the symptoms grow worse, and he is compelled to take to his bed. He then complains of a sense of chilliness, and sometimes of a severe chill, followed by fever and a dry, husky skin.

The temperature of the body ranges from 98.5° Farh., in the morning, to 100.5°, in the evening; the pulse is from 90 to 110 per minute; the appetite is entirely lost; the bowels move more frequently; and the temperature continues to rise to 101.5°, in the morning, and to 104.5°, in the evening, as the disease advances. If the temperature rises to 105°, the disease is of a grave character; and if it still goes up to 106°, the patient is in great danger, and generally dies.

The tongue becomes thickly coated; sordes gather around the teeth; the countenance assumes a dull expression; a low, muttering delirium sets in; the patient is nervous and has a buzzing in the ears, which so affects the hearing as often to produce deafness; he sometimes doses, and starts up in a state of alarm; he is easily aroused, and answers questions, but soon relapses into a somnolent condition. If the abdomen be examined closely, between the seventh and fifteenth day of the progress of the disease, a few red spots will generally be found upon it, which may extend to the breast, and also to the extremities. This is called the rose-colored eruption, and is one of the characteristic signs of typhoid fever.

As the disease advances, the patient becomes delirious; the fæces are passed involuntarily; he becomes comatose, picking at the bedclothes or imaginary objects; slipping down in the bed; the skin is bathed with a clammy sweat; the pulse grows quick and feeble; subsultus tendinum is observed; and death often takes place in from six to twenty-one days, from the time the patient takes to his bed. Although this is the course of the fatal cases, yet a large majority of the patients, if seen in time, and the appropriate treatment adopted, begins to improve about the 9th, 14th, or 21st day. The tongue begins to clean from the tip and edges; the pulse grows less frequent; the skin is cooler; the mind becomes clear; the patient sleeps, awakes, and finds himself refreshed; the bowels return to their normal function; there is a desire and relish for food; and convalescence is established.

The tongue, sometimes, instead of cleaning from the tip and edges, throws off its coat in flakes from the centre. In this condition, the prognosis is favorable, but the convalescence is more tedious.

This, then, is the beginning, progress, and termination of simple, uncomplicated typhoid fever. But a more dangerous variety often prevails, or the simple may merge into, or put on, a more violent form, which is denominated—

### TYPHO-ENTERIC.

This differs from the former in the degree of lesion in the intestine only. In this variety, the diarrhœa is the first and most constant symptom. The patient complains of tenderness of the bowels; the stools are frequent, thin, and watery, and of a brownish cast; the abdomen becomes tympanitic, with a gurgling sound in the right iliac region, when pressure is made upon that part; the urine is scanty and highly colored, and is sometimes suppressed or retained in the bladder.

The tongue, which at first was covered with a thin, whitish fur, now becomes brown and dry in the centre, with a thicker coat; the tip and edges are of a crimson hue, and sordes gather around the lips and teeth. As the disease progresses, the abdomen becomes more distended; the stools are more copious and contain dark, fœtid blood, which is an evidence of disintegration of the structures of Peyer's patches. Sometimes the hemorrhage is so great, that the patient is prostrated in a few hours; but, more generally, ulceration of the coats of the ilium takes place gradually, with increased tenderness of the bowels; and after a longer or shorter continuance of these symptoms, the patient is suddenly seized with a violent pain in the right iliac region; he looks distressed and anxious.

These symptoms point to perforation of the intestine, with extravasation of its contents into the peritoneal cavity, which produces inflammation of the peritoneum, and may cause death in from one to six days. However, ulceration and perforation of the ilium does not always cause death. I am a living evidence of this fact; and there are many cases of recovery on record.

During the fall of 1865, I was the subject of an attack of typho-enteric fever; and from the large amount of fœtid blood, and the character of the fecal matter that passed from the bowels, and from the locality and severity of the pain and tenderness, there was unmistakable evidence of an extensive ulceration, if not perforation, of the intestines. This was the opinion of L. Powell, M.D., a talented physician of Louisville, who was so attentive to me during my long and tedious illness. For six months after I became convalescent, I never had a free and easy stool. The right side of the abdomen was tender and felt contracted, and there seemed to be some obstruction to the free evacuation of the bowels. The fæces gave evidence of having passed through a narrow space. Those conditions were, no doubt, the result of cicatrices, which caused contraction of the muscular coats of the intestine, and thereby diminished the calibre of the bowel, through which the fecal matter had to pass.

There was one other peculiar and very distressing symptom in my case. After I became conscious, I had a feeling as if my body had been severed at the umbilicus. This condition lasted about ten days, when sensation of the extremities began to return, and with it the most intense suffering that I ever experienced. If a thousand needles had pierced my flesh, they could hardly have been more painful. For two months, I suffered more or less from that pricking sensation, and did not regain my usual gait for 12 monts or more. I regarded that partial loss of sensation and of motion as an evidence of the effect produced on the cerebro-spinal system by typhoid poison.

The next variety in the catalogue of complications is the cerebral type, or—

TYPHO-CEREBRAL.

In this, the head symptoms are more marked than in any other. The patient complains constantly of headache, from the incipiency of the disease; and as the fever and temperature of the body increases, he becomes wild and excited, and is soon delirious and talking incoherently. The head is sometimes hot and dry; the patient cannot sleep, and imagines that he is away from home, and is constantly arising from his couch, and attempts to make his escape through an open window or door, or sometimes falls helpless on the floor from exhaustion.

The question naturally arises, whether the head symptoms are the result of inflammation, or of functional derangement. It is believed that the latter is the pathological condition, in the majority of cases. For while persons have died, apparently, from the effect of the cerebral symptoms, *post mortems* proved the fact that the brain and its appendages were free from inflammation, and that the characteristics of typhoid fever were manifest in the ilium; thus proving, beyond a doubt, that whatever may be the complication or symptoms, the specific agent by which the system is depressed, after changing the condition of the blood, selects the glands of Peyer as the point from which its influence is communicated to the various organs and tissues, either directly through the circulation, or by reflex action.

I do not wish to be understood as saying, that there are no cases of inflammation of the brain and its meninges ever found in typhoid fever; for there are exceptions to all general rules. But, in this disease, depression of the functions is the rule, and inflammation is the exception. Aside from the evidence furnished by *post mortems*, we may reason from a therapeutical point of view to prove this proposition.

The books are unanimous in condemning the use of opium in cerebral meningitis. They declare that it is not only contra-indicated, but is positively injurious. But, on the other hand, every practitioner of medicine is aware of the happy effects of two or three grains of opium, in coma-vigil and typho-mania, of the typho-cerebral type of typhoid fever. So, then, the cerebral symptoms of this disease are not due to inflammation, or, if they are, then opium is the remedy *par excellence* for meningitis. But I do not see that there is anything to be gained by discussing this subject; for it is evident that the typhoid poison is capable of so depressing the nervous centres as to arrest the organic functions, and destroy life as surely as if inflammation had existed.

According to my observation, the inflammatory or non-inflammatory character of the cerebral symptoms, in typhoid fever, may be established by the thermometer. In this disease, the temperature varies in accordance with the degree of activity of the typhoid poison. There is always a difference of from 1° to 2° F., between the evening and morning temperatures; and this difference is always observed, whether the evening temperature is rising or falling. The temperature gradually rises in the evening and falls in the morning, until the former reaches 104°, and the latter 103°. When the temperature has reached these points, which is from the third to the sixth day, there is a decrease, and it often falls as low as 99.5°, in the morning, and 100°, in the evening. This fluctuation continues for a longer or shorter period, when the temperature may again go up, until it reaches 105°, in the evening, and 103° in the morning. Thus the fluctuations take place from time to time, and the oscillations of temperature, between the evening and morning, continue with the same degree of regularity, until the beginning of the stage of convalescence, when the difference of oscillation may reach from 3° to 5°. But, on the other hand, in local inflammations, there is a gradual rise of the temperature, until the inflammation has reached its acme. And as the inflammation begins to decline, the temperature falls rapidly, until the standard of health is again reached.

The following are notes of a well-marked case of typhoid fever, of the typho-cerebral type. John Riccius, aged eight years, nervous temperament, had never been sick before; was troubled with headache, constipation, loss of appetite, sleeplessness, and general languor, from March 4th until the 10th, when he was confined to bed, with an aggravation of all the symptoms just mentioned. At 7 P.M., his countenance was expressive of pain and nervous excitement, with pain and tympanitis of the abdomen, and gurgling in the right ileo-cæcal region; stools thin and watery; skin was hot and dry; tongue covered with a brown fur; temperature 104.5° Farh. The temperature was first taken on the sixth day of the disease, and on each subsequent day of its progress, as shown by annexed table:—

It will be observed, that on the 10th, the thermometer showed a rise of temperature, in the evening, to 104.5°; from the 10th to the 14th, the temperature had a downward tendency, until it fell to 99.5°. It oscillated until the 16th, when it rose to 104.5°, in the evening, and fell again to 101°, on the morning of the 17th, and rose again to 105°, in the evening of the same day. From this time until the 24th, the evening and morning temperature oscillated between 105° and 101°, when it fell to 99°, but arose again to 103.5°, in the evening. Thus the fluctuations continued until the eighth day of April, when the temperature fell to the normal standard, and the patient gradually recovered.

RANGE OF MORNING AND EVENING TEMPERATURE, FROM MARCH 10TH TO APRIL 8TH.

CHICAGO MEDICAL EXAMINER,

*June, 1870.*

[To face page 328.]

The reason that there is not so great a lesion of the ilium, in the typho-cerebral type of typhoid fever as there is in the typho-enteric is, because the force of the disease is called from the bowel to some distant organ. And there it may exert such an influence over the organic functions, as to increase susceptibility, destroy vital affinity of the parts, and produce death, by depression of the vital powers. A somewhat common and very dangerous complication is called—

TYPHO-GASTRIC.

Soon after the characteristic symptoms of typhoid fever have been developed, the patient complains of nausea and vomiting, and of a load and pressure in the epigastrium. When vomiting occurs, it is only an ejection of the fluids that have been taken into the stomach. The fluids that are thrown up are sometimes of a yellowish cast, owing to regurgitation of bile. The pulse is quick and full; the skin is dry and hot; the tongue is covered with a dirty-white fur, and is a little brown in the centre. This form of typhoid fever is the most dangerous; and it may be mistaken for gastritis. It frequently destroys life in a few days. Owing to the fact that there is such a strong impression made upon the stomach, the bowels are not so loose as they are in the other varieties; and thus we may overlook the intestinal symptoms. If, however, they be examined carefully, the tenderness may be detected near the ilio-cæcal valve.

Another variety of typhoid fever often occurs in certain latitudes during the winter and spring, which has been named—

TYPHO-PNEUMONITIS.

I have placed typhoid-pneumonia in the classification of continued fevers, because the idiopathic fever precedes the inflammation of the lungs; and the pneumonia is symptomatic, and, therefore, secondary to, and a complication of, typhoid fever. Whereas, in pneumonia proper, we may have a typhoid condition supervening the disease of the lungs; therefore one is the result or consequence of an idiopathic fever, and the other the effect of a primary local lesion.

This disease has frequenly prevailed as an epidemic, in some portions of the United States; and it is sometimes attended with very fatal results. West and central Virginia are localities where it is frequently seen during epidemics of typhoid fever. After the usual premonitory symptoms of typhoid fever have lasted for a longer or shorter period, the patient complains of a sense of chilliness, followed by a dryer and hotter skin; the secretions are locked up; the tongue is dry and of a brownish cast; the face is flushed and of a bluish tint; the pulse is strong, full, and rapid; the countenance is dull and the respiration is quick. This latter symptom is peculiar to pectoral troubles, and calls our attention to a complication of pneumonia.

If the ear be applied to the chest, a crepitant rale may generally be heard in the lower and posterior lobe of the right lung; there is but little or no pain, and but little cough in the early stage. If there is any pain, it is dull, and not the sharp pain of pneumonia proper. Sometimes the symptoms are so slight, that the complication may be overlooked without the aid of auscultation. But as the disease advances, the force of the idiopathic fever seems to be concentrated on the lungs; and we have cough and difficulty of breathing, as in primary pneumonia; but there is a greater degree of prostration.

The last complication of typhoid fever to which I call attention is—

TYPHO-MALARIAL FEVER.

This disease prevails endemically and epidemically in malarial districts only, and partakes both of typhoid and malarial fevers. After the usual though aggravated symptoms of typhoid fever have lasted from three to seven days, the patient complains of a cold sensation or severe chills, with aching of the back and limbs; the tongue becomes coated in the centre with a thick, brown fur; the edges and tip have a glossy, red appearance. The bowels may be inclined to constipation or diarrhœa, but generally the latter. The patient is nervous, and as the disease approaches its acme, delirium sets in, and he soon sinks into a profound stupor, and death often takes place, in from seven to fifteen days after the chills make their appearance.

There is one peculiarity about this disease that may lead the physician into error in diagnosis. The patient is found perspiring freely, and has all the appearance of having a malarial fever; he complains of being cold, even while in this condition; and the pulse is never less than 110, and may reach 140, while he is sweating profusely; the tongue is still dry, in this stage, and the temperature is not reduced. It has been observed that the cases which perspire copiously are the most dangerous and difficult to treat. As a general rule, if the patient perspires freely, the diarrhœa is easily controlled; but when the skin becomes dry, the bowel affection is very troublesome. The gastric and cerebral functions are generally deranged, in this form of the disease; nausea and vomiting are almost always present. There is no regularity in the appearance of the rose-colored eruption; it may show itself on the seventh day, and from that to the twenty-sixth. I have generally found audamina over the body, instead of the rose-colored eruption on the abdomen.

Some writers have described another variety, which comes on suddenly, with chills and remissions, similar to remittent fever, and followed by typhoid symptoms. I am inclined to believe that those are aggravated cases of remittent fever, which merge into a typhoid state. For in the cases which I have seen, there seemed to be a comingling of the symptoms of typhoid and remittent fever, throughout the whole course of the disease—that is to say, the primary and general symptoms were characteristic of typhoid, and the incidental were those of remittent.

During the summer of 1863, I treated seven cases in the northern part of Illinois. They were well-marked cases of typhoid fever, in the beginning, and continued so through the first five or six days, after which the patient complained of chilly seneations, followed by a hotter and dryer skin. The pulse would often reach 140 per minute. This febrile excitement would sometimes continue for 12 or 18 hours, when the patient would break out into a copious perspiration. I have seen patients perspire freely for 24 hours, and then the skin would become hot and dry for the next 24 or 30 hours. I noticed that during the most profuse sweatings, the patients complained of being chilly, and would draw the covering around their necks. The pulse would fall only a few beats in this stage. The patients were inclined to be drowsy, but were easily aroused, and would soon relapse into a dreamy or half conscious state.

The apparent regularity of the sweating stage, and the character of the perspiration, gave evidence of the presence of marsh miasmata in the system. But every other symptom pointed unerringly to a typhoid poison. One of those patients died, and the otheis were from 14 to 21 days before convalescence was established.

The liver is frequently deranged in this disease. The fluids ejected from the stomach are often colored with bile, and the conjunctiva has a yellowish cast.

Hiccough is often a troublesome symptom, and is much more frequent in this, than in the other varieties of typhoid fever.

*Causes.*—The cause of typhoid fever is not well understood. There is no doubt that a specific agent is formed by the decomposition of animal matter, or a chemical change takes place in the exhalations from the human body, during sickness or close confinement, which acts as a predisposing cause. Thc exciting causes are vicissitudes of climate, errors in diet, mental anxiety. and whatever tends to increase susceptibility of the system, and induce that peculiar condition called the aplastic diathesis. When the disease occurs under these circumstances, and is confined to particular localities, it is said to be endemic.

The epidemic form may be produced by an electrical charge in the atmosphere, by which the vital powers of the inhabitants of cities and large territories are so depressed as to form a favorable medium by which the specific agent readily diffuses itself through wide districts; and all those who are affected by the predisposing cause, are easily brought under the control of the exciting cause, and are prostrated by the fever.

The morbific agent, whatever it may be, does not affect or produce zymosis in the plastic system; and the individual must first be brought under the epidemic influence, or his condition changed, before he is liable to an attack of typhoid fever.

If, in certain geographical districts, the atmosphere becomes damp and heavy, with thick fogs remaining during the greater part of the day, and continues for several days together, typhoid fever will be the prevailing disease, and will give all the evidence of an epidemic.

This disease is thought to be most prevalent in autumn and winter. If it prevails in malarial districts, during the latter part of summer or early autumn, it is liable to put on a typho-malarial type.

It appears that no age or sex is exempt from typhoid fever. From the investigations of Louis, it is found to be more frequent in those from twenty to twenty-five years of age. But it frequently occurs in children, at the age of eight and nine years.

There are many persons who believe this disease to be contagious, but some of the ablest practitioners of Europe and America discard the theory. There is no doubt that the idea originated from the fact that it is sometimes difficult to draw the line of distinction between typhus and an epidemic of typhoid fever.

There is no positive evidence that typhoid fever is contagious. It was so considered by the older authors, but it was because they were unable to draw the line of distinction between typhoid and typhus. Since the days of Louis, the distinguishing characteristics have been made plain. The fact of several members of the same family having typhoid fever, is no evidence of its being contagious. Neither is the fact of persons visiting the patient from a healthy locality, and then returning home to have the disease, an evidence of their having contracted it by contagion—unless we call all causes of disease contagion. It simply proves that they remained long enough under the influence of the local cause to change their diathesis, and place them in that condition which is susceptible of endemic diseases.

Then again, the care and anxiety manifested by the family for the sick member, loss of sleep, bad ventilation of his room, and the constant breathing of the exhalations from the patient, are predisposing causes; and any person being kept in that condition for any length of time, must evidently take the fever, or undergo a pathological change of a typhoid character.

But if the patient is kept clean, and his chamber thoroughly ventilated night and day, there is no danger of any one contracting the disease by visiting him. For the system must first be brought under the control of the same endemic or epidemic influence, before it can be attacked by the same form of disease.

But, on the other hand, contagion is liable to attack any person, with the smallest amount of exposure. There is then a wide difference between the virus of contagion and the agent of endemic and epidemic diseases. And until we are able to make this distinction, we are liable to commit an error in regard to the contagiousness and non-contagiousness of certain diseases.

*Morbid Anatomy.*—The almost constant pathological peculiarity of typhoid fever is to be found in the ilium, as the result of the fever poison.

An irritation and congestion are set up in the glands of Peyer, which give them a thickened or enlarged appearance; and as the system is fully brought under the influence of the idiopathic fever, inflammation sets in; disintegration takes place; the glands become softened, and easily break down; the follicular structure is lost; and ulcers of varying size and color are the result.

These ulcers have a tendency to heal even where perforation has taken place, and under appropriate treatment, do frequently heal, and the patient recovers. This fact has been proved by *post mortems*, where patients have died of some other disease, many years after an attack of typhoid fever.

It would be natural to suppose that, from the general effects of the blood poison, and the long continuance of the disease, the various organs and tissues of the body would undergo a pathological change before death: this we find to be the case. The liver, spleen, and mesenteric glands are found softened and enlarged; the kidneys are in a morbid condition; the pharynx, œsophagus, bronchi, lungs, stomach, and heart are more or less changed in color and structure.

### DIAGNOSIS.

The diagnosis of this disease is sometimes difficult, owing to the fact that many of its symptoms are similar to those of other idiopathic fevers, and simulate those of local disease and morbid states. There are, however, some differential symptoms which serve and direct us to a correct conclusion.

The watery evacuations and tympanitic bowels; tenderness over the iliæ region, with a gurgling sound; the thin, dirty white fur on the tongue, which after a few days becomes brown; the rose-colored rash on the abdomen; complete loss of appetite; the peculiar dulness of mind, and apathetic or dejected expression of countenance; and enlargement of the spleen are all diagnostic symptoms of typhoid fever, when taken in connection with the history of the case. The latter symptom is supposed to be pathognomonic, if it can be shown that the spleen was not enlarged previous to the attack then under consideration.

The diseases and conditions most likely to confuse the physician are the following:—

| | | |
|---|---|---|
| Typhus fever, | Enteritis, | Typhoid condition, |
| Remittent " | Gastritis, | General Debility. |
| Meningitis, | | |

TYPHUS.—The differential diagnosis of typhoid and typhus fevers may be summed up as follows:

| TYPHOID. | TYPHUS. |
|---|---|
| 1. Forming stage from one to ten days. | 1. Forming stage from one to three days. |
| 2. Diarrhœa. | 2. Constipation. |
| 3. Susceptibility to the action of purgatives. | 3. No tendency to diarrhœa, or excessive purgation from purgatives. |
| 4. Countenance pale, or of a reddish tint. | 4. Countenance of a dusky hue. |

| | |
|---|---|
| 5. Abdomen tympanitic, with tenderness, and a gurgling sound upon pressure in the right iliac region. | 5. Abdomen flat; and if there is tenderness, it is general, and not confined to the right iliac. |
| 6. Coffee-ground stools. | 6. Dark, offensive stools, but not watery. |
| 7. Hemorrhage from the bowels frequent. | 7. Hemorrhage from the bowels seldom. |
| 8. Rose-colored eruption—disappear upon pressure. | 8. Petæica of a livid hue and but little affected by pressure. |
| 9. Ulceration of the ilium is a constant pathological peculiarity. | 9. No constant pathological peculiarity. |

*Remittent Fever.*—This fever may sometimes baffle us in our diagnosis, unless we are on our guard. But if we remember that periodical fevers come on abruptly, and with great severity, and that typhoid fever begins slowly, and gradually increases in severity, and is always accompanied with diarrhœa, or a predisposition to it, we need not have any doubts as to the character of the affection.

And then, again, there is a decided remission and exacerbation in intermittent fevers; sometime during the twenty-four hours; whereas in typhoid the fever is continuous, except in that variety which we have designated as typho-malarial, in which there seems to be a blending of the symptoms of the two. But generally, if we have a correct knowledge of the clinical history and symptoms of these diseases, the careful observer need not make any mistake.

*Meningitis.*—The symptoms of the typho-cerebral type of typhoid fever and meningitis may be mistaken for each other, unless the practitioner is familiar with the characteristics of the two affections. But the suddenness of the attack; the flushed face; the quick, full, and bounding pulse; throbbing of the carotid and temporal arteries; the sharp pain in the head, with convulsive movements; the injected eyes, and constipation of the bowels all distinguish the latter from the former.

*Enteritis.*—This disease may be distinguished from typhoid fever by remembering that in the former, the inflammation of the intestines is the primary disease, and precedes the fever, and the inflammatory action is more extensive over the abdomen; the pain and tenderness is more acute and constant in the early stage; and the bowels are costive. While in the latter, the fever is primary, and the morbid condition of Peyer's glands is secondary; and the tenderness and pain are local, and confined to the ileo-cæcal region; and the bowels are loose from the beginning.

In enteritis, there is but little prostration, until the disease has progressed for some time, or passed into the typhoid state. The spleen is normal; and there is no mental wandering, no rose-colored spots on the abdomen, nor sudamina on the body: while in typhoid fever the reverse is true.

*Gastritis.*—Unless the physician is on his guard, a dangerous form of typhoid fever may escape his observation, or be obscured by gastric derangement, and the patient may be lost before he is aware of the nature of the malady.

Functional derangements of the stomach are not accompanied with febrile excitement. But gastritis and typho-gastric are followed and preceded by fever. In the former, the fever follows as a result of the inflammatory process set up in the stomach, pain and tenderness being the first symptoms manifest to the patient; and after a longer or a shorter period, the fever is developed. In the latter, the fever is the primary symptom, and the gastric disturbance is secondary.

Nausea may be one of the earliest symptoms in the forming stage of typhoid fever; but tenderness and excessive vomiting are not experienced until the fever and temperature begin to rise.

Gastritis may generally be traced to some error in diet, or the act of swallowing some irritating substances: whereas, the history of the case, its gradual approach, with all the phenomena that accompany continued fevers, will point unerringly to typho-gastric.

General Debility simulates typhoid fever in its prostration of the organic functions, but the pathognomonic symptoms of the latter are wanting in the former; and by gaining a correct history of the case, and tracing the debility to its source, there need be no error committed in diagnosis.

*Typhoid Condition.*—It has ever been a source of annoyance to the young practitioner to trace the boundaries which separate typhoid fever from a typhoid condition. But if the distinction be firmly fixed in the mind that the former is a primary disease, and that the latter is a secondary morbid state, there need be no difficulty on this point. A typhoid condition may supervene an attack of any disease; therefore, our first duty is to trace the case to its origin, weigh every symptom and see whether they belong to a primary disease or a secondary state. If the clinical history and symptoms of the case point to the former, then what is the character of the affection? is it a general or local trouble? This question can only be satisfactorily settled in proportion to our knowledge of the symptoms and peculiarity of those diseases, and our capability of weighing and sifting testimony.

As we take our seat by the bedside of the patient, the question often arises whether it is a case of typhoid fever, or is it a typhoid condition? If, in tracing the history of the affection, we find the symptoms and peculiarities of the former present, then we know that the case is one of typhoid fever. But if, on the other hand, the historical and most prominent symptoms of that disease are wanting, we infer that the morbid change is one of a typhoid condition, and is the result of some other malady, which must be diagnosed in accordance with the primary symptoms.

*Prognosis.*—The prognosis of this disease is generally favorable; a large majority of the cases recover with proper sanitary regulations. Yet it should be guarded against; for there is no case so favorable that it may not disappoint us. In the midst of convalescence, when all the symptoms point to a speedy recovery, some untoward occurrence often takes place, and the patient sinks in a few hours.

The unfavorable symptoms are an increased frequency and feebleness of the pulse, a comatose condition of the patient, involuntary discharges, subsultus, contraction of the muscles around the mouth and nose, picking at the bed clothes or imaginary objects, slipping down in the bed, and a cold, clammy sweat; all point to a speedy dissolution of the patient.

The indications of a favorable issue and a speedy convalescence are, a diminution in the frequency of the pulse, a gradual fall in the morning and evening temperature, the skin becomes cooler, consciousness returns, the tongue begins to clean from the tip and edges, the secretions return to their normal condition, the tympanitic abdomen begins to subside, and there is a returning relish for food.

TREATMENT.

The treatment of typhoid fever has been, and is still, a subject of discussion. The four methods of treatment, or the therapeutics advocated by some of the older writers and physicians, and many of the present day, are venesection, brandy, emetics, and cathartics.

A moment's reflection will suffice to discard them all. And first, by bleeding, we draw off the very materials which we most need to sustain the patient, to build up the tissues, and to carry him safely through the future progress of the disease. Furthermore, it has been demonstrated that we cannot shorten the progress of the disease by venesection, but rather prolong the cure. I do not deny that bleeding has done good in typhoid fever of the sthenic grade, by checking the flow of blood to the parts, and thereby preventing or arresting inflammatory action. But I should not like to risk the prostrating effects of venesection, when we have a sure sedative in the veratrum viride and gelseminum; and especially as they do not exhaust the elementary properties of the tissues like bleeding.

There are many persons who advocate strongly the use of brandy or alcoholic stimulants, in typhoid and typhus fevers. But we have only to compare the pathological condition of the blood and vital forces in those diseases, and the physiological effects of alcohol, to show the impropriety of its use in those fevers.

Experimenters are agreed that the blood, in typhoid and typhus fevers, is imperfectly decarbonized; the fibrin is impaired in its coagulability; the functions of the nervous and muscular tissues are greatly depressed; susceptibility is increased; vital affinity is diminished; and the plasticity of the blood in a measure destroyed. Alcohol, while in the human system, diminishes the decarbonization of the blood; it retards the coagulability of the fibrin, produces an anæsthetic or depressing effect on the nervous centers, and diminishes organic changes (Davis).

The first or temporary effect of alcohol on the system is that of an arterial stimulant, and its secondary effect is that of a powerful sedative. All investigators agree that alcohol is not digested, when taken into the stomach, and cannot therefore act as food, but passes through the circulation, and is thrown out of the system as a foreign substance; not, however, until after it has left its fearful inroads upon the tissues and organic functions.

With this view of the subject, I discard almost entirely the use of alcoholic liquors in my practice, especially in low forms of disease; and I have never yet found occasion to regret it.

In a lecture of W. T. Gairdner, Professor in the University of Glasgow, he said that he did not object to the moderate use of alcoholic liquors in certain stages of typhoid and typhus fevers. But he condemned its indiscriminate use in such strong terms, and backed his opinion with such powerful reasons, as to convince any one that it is not only contraindicated, but that it is positively injurious in almost every stage. I quote from Braithwaite, Part 51, Page 22. He said:—

"To give wine, whiskey, or beef tea, while withholding milk, is simply, in my opinion, to destroy your patient; and the more wine or whiskey you give, while withholding milk, the more sure you will be to destroy your patient soon, because you are thereby superseding the natural appetite (or what remains of it) for a nourishing and wholesome diet, by a diet—if it can be so called—which so prisons the blood, and checks the secretions, and alters for the worse the whole tone of the nervous system, and of the digestion and assimilation. I believe that infinite mischief has been done in typhus fever, and in all fevers, by giving wine, and withholding or not giving milk." And further along he remarks that "You must absolutely make up your mind to feed your patient naturally, and not to stimulate him."

Emetics and cathartics have their advocates in typhoid fever. The former sometimes do good by unloading the stomach of undigested food, or accumulating and irritating fluids. And they may do good by arousing the secreting organs to action, by the muscular contraction produced in the act of vomiting. But, upon the whole, they are uncalled for, from the fact that the appetite is impaired from the beginning of the disease, and by the time that the physician is called, the stomach contains but little food.

Cathartics should only be mentioned, to be condemned; and they have justly passed under the condemnation of all intelligent practitioners. One free catharsis may prolong the disease from one to two weeks, or it may kill the patient. I learned this lesson by sad experience. The first case of typhoid fever that I lost, occurred under the following circumstances: I had been closely watching the patient for ten days or more, to arrest the diarrhœa. I succeeded in checking it, and the patient seemed to be convalescing. The pulse was nearly normal, the temperature came down rapidly, and the patient had some relish for food. The friends became anxious about the bowels, and desired the patient to have a stool. I put them off by telling them that I would order an injection when it became necessary. But they were not satisfied; and at the end of thirty-six hours after the diarrhœa had been checked, they prevailed upon me to give her a dose of castor oil. After six hours, the oil operated mildly; in one hour more she went to stool again, and had a copious evacuation, containing fœtid blood. The motions became so frequent, thin, watery, and bloody, that I was sent for, fifteen hours after the oil had been taken (she lived six miles in the country, consequently I visited her but once a day). When I reached her bedside, I found her cold to the knees and elbows—the pulse was so quick that it could not be counted. She died, not directly of typhoid fever, but of catharsis, and that, too, within twenty-four hours after the administration of the oil. A befitting epitaph would have been,—"Died by one table spoonful of castor oil."

It may be said that the fœtid blood was accumulating, and would have burst forth sooner or later. That may be true, but I am satisfied that a table spoonful of castor oil broke away the checks which I had placed upon the disease, and carried my patient into eternity. I would therefore urge upon the practitioner to be careful how he gives cathartics, in the typho-enteric variety of typhoid fever. If, however, we fear that there is any irritating matter in the stomach and bowels, at the beginning of our treatment, it will be well to give a mild laxative or an enema, followed, after its operation, by opium, so as to prevent excessive purgation.

One of the most important items to be observed in the treatment of typhoid fever, is the proper regulation and promotion of the hygienic agents, *viz.:* air, aliment, excretions, sleep, cleanliness, and affections of the mind.

Without pure air and a free ventilation, our best efforts in medication will prove abortive. In typhoid fever, the patient's apartment should be ventilated both night and day. It is not only necessary to have windows let down from the top, but a free draught of air should constantly be made to pass through the room, night and day, so as to carry out all the exhalations from the patient. It is not necessary that the draught should blow directly on the patient, nor is it always safe, except in warm weather; then the patient may not only lie in the draught, between two doors or windows with impunity, but with benefit. But if the weather be damp or cold, it will be best to screen the patient from the direct influence of the air, and kindle a little fire in his chamber.

Cleanliness is another important consideration in the treatment of typhoid fever. When the patient is feverish, and the skin is dry, his body should be sponged once a day, or oftener, with cold water, containing chlorate of potash; and the linen of his person and bedding should be changed every day. The excrements should be immediately removed from the room, and the apartment purified, in addition to free ventilation, by some antiseptic. In a word, the patient's bed chamber should, if possible, smell as fresh and pleasent as a drawing-room. Only in this way can we hope for a quick and favorable action of our medicines, and a speedy convalescence of our patient.

The diet of a patient with typhoid fever is a subject of the highest importance, both in its character, mode of preparation, and its use. It must be nutritious, small in quantity, and unirritating in quality. In all the varieties of this disease, the milk porridge is the best diet that can be given. It is nutritious, pleasant, and agreeable to the stomach. Rice boiled in milk is palatable and easy of digestion; but where we require an organic stimulant as well as nourishment, there is nothing equal to beef-essence, well salted. We get a large amount of nutriment in a small bulk of material. The best way to prepare the essence is to chop fine a piece of fresh tenderloin of beef, freed from fat, salt, and put into a bottle without water, cork loosely, set into a kettle of water so that the mouth is not covered, and boil until the beef is cooked tender. The fluid, as it collects in the bottle, should be poured off into some suitable receptacle, from time to time, so as to prevent the evaporation, during the process of preparation.

As convalescence progresses, soft boiled eggs may be allowed in moderation, but the patient's return to solid food must be gradual, and with great care.

The excretions, sleep, and affections of the mind, which are under the control of physiological laws and the will, and which are so conducive to health, are so morbidly affected in this disease, that the patient loses his power of regulating them; and the secretory, excretory, and nerve functions are so perverted, that remedial agents are necessary to assist nature in restoring the organs to their normal action.

The indications for treatment in this disease are: 1. To lessen febrile excitement and allay morbid heat. 2. Promote the excretions. 3. Antidote or neutralize the fever poison, and eliminate the effete matter from the system. 4. Overcome morbid susceptibility and irritability. 5. Arrest and heal local lesion. 6. Increase the vital affinity and tonicity of the system.

The first indications may be fulfilled by fresh air, cold spongings, and sedatives, the best of which are veratrum viride and gelseminum. Secondly, the secretions are more readily promoted by alterative doses of calomel and Dover's powder. Thirdly, the best antidotes for the poison are the sulphite of lime and soda, chlorate of potash, and muriated tincture of iron. The effete materials may be eliminated through the kidneys, skin, and lungs, by diuresis, diaphoresis, and whatever promotes a free ingress of pure air into the lungs. The lung tissue absorbs the oxygen of the air, and imparts it to the blood, which in turn gives off its carbon and other impurities, and at each act of expiration, they are thrown out of the system, Fourthly, increased susceptibility and irritability may be overcome by nervous sedatives and anodynes, of which, some of the preparations of opium and chloroform are the best. Fifthly, to correct local inflammation and ulceration, we use anodynes, alteratives, local stimulants, and astringents. Sixthly, vital affinity and tonicity of the system are to be promoted by tonics and organic stimulants.

Having noticed the general principles to be observed in the treatment of typhoid fever, I now propose to take up the special treatment of its varieties, in the same order that I discussed their symptomotology.

*Treatment of Simple Typhoid Fever.*—First then, the treatment of simple typhoid fever must be adopted in accordance with the severity of its symptoms. If it is of the sthenic grade, we may begin by giving the following sedative mixture:

℞. Etherus spiritus nitrici, ------------------ ℥iss.

Opii tinctura camphorata, ------------------ ℥ss.

Tinct. veratri viridis, ---------------------- ʒss.

Mix, and give a teaspoonful every four hours, until the fever abates. As an alterative, give one grain of calomel and five grains of Dover's powder three times a day. We continue this treatment until the secretions are promoted, and the skin becomes cool and moist. Calomel should never be given so as to affect the mouth, in typhoid fever; therefore, as soon as the tongue becomes moist, and the secretions are established, dispense with it. Then put the patient on the use of thirty grains of sulphite of lime or soda, and one-sixth of a grain of sulphate of morphine, three times a day. If the bowels are very loose, it will be best to use the lime, but if they are not, the soda may be given.

While the head and body are hot and dry, sponge the former frequently with cold water, containing chlorate of potash, and the latter twice a day. His linen should be changed once a day, and, if the weather is warm, open the doors and windows, and leave them open night and day. If the weather is cold, cover the patient up warm, keep fire in the room, and ventilate it freely.

If the bowels become costive, a table spoonful of castor oil may be given, containing ten drops each of spirits of turpentine and laudanum. Directions should always be given to have the bowels checked after the first stool, by opium or otherwise, for running of the bowels may convert the simple type of fever into the typho-enteric, and thereby increase the danger of the patient.

The drinks of the patient should be cold. Ice or ice-water, lemonade, and the effervescing draught are refreshing, and may be taken *ad libitum.* The latter is especially beneficial, if there is nausea, and the skin is hot and dry.

*Asthenic Grade.*—If the pulse is quick and feeble, with a general depression, we do not give the sedative mixture, but we give the spirit mindereri, and the alterative. We continue the calomel and Dover's powder, as before, until the secretions are established. We then discontinue the alterative, and give the one-sixth of a grain of morphine, and ten grains of chlorate of potash, three times a day, alternated with thirty grains of the sulphites.

*Diet, &c.*—In the treatment of typhoid fever, there is probably nothing of more importance than the proper regulation of the patient's diet, and a free ventilation of the sick chamber. The patient must be sustained by nourishment and tonics, in order to enable the constitution to combat the ravages of the disease. Animal broths, well salted, and milk are to be used freely. Milk porridge is the very best diet that can be given in this and all other low forms of fever.

As tonics, the mineral acids, tincture of prickly ash, and quinine in small doses, are the best. The utmost care must be taken both as to exercise, and a return to solid food. Many persons have passed through a long and tedious attack of typhoid fever, and finally brought on a relapse and died, from overloading the stomach with solid food.

*Treatment of Typho-Enteric.*—The treatment of this variety must be conducted upon the same general principles as that of simple typhoid fever, for the former is only a higher grade of the latter, or it has a more local character. Therefore our especial attention must be directed to the pathological condition of that portion of the ilium occupied by Peyer's patches. Our first effort should be to check ulceration of the intestines, and thereby prevent death by perforation. In order to accomplish this, we must arrest the morbid secretion and action of the bowels; for the constant passing of acrid secretions over the inflamed mucous membrane of the intestines, increases the inflammation, and hastens ulceration. By far the best agent now at our command is the turpentine emulsion, made as follows:

℞. Oleum, Terebinth, ---------------------------- ʒij.

Pulvis Acacia, }

Sacchar. Alba, } āā, --------------------- ʒiij.

Aqua Mentha, ---------------------------- ℥ij.

Tinct. Opii, ------------------------------- ʒij.

Mix, and give a teaspoonful every two to four hours, until the bowels are checked; then lengthen out the dose to intervals of every six or eight hours, until tenderness has subsided. We give the turpentine emulsion in the early stage, not only to arrest the diarrhœa, but it is an invaluable remedy in the ulcerative stages of Peyer's glands. The turpentine penetrates the ulcerated surfaces, corrects the morbid action, stimulates the tissues, and sets up the healing process. The tincture of opium is beneficial in arresting the peristaltic movements of the bowels, and allaying morbid irritability and susceptibility of the parts; and by its power of arresting secretion it promotes resolution.

At the same time that our attention is directed to the bowels, the general circulation must be watched; and if there is high arterial action, the sedative mixture, containing veratrum, must be given as before directed. But if the asthenic grade be present then the effervescing draught, or the spirit mindereri, should be given. And in either case, the alterative of calomel and Dover's powder must be given, until the tongue becomes moist, and the portal circulation is unlocked.

If the turpentine emulsion fails to check the ulcerative process, or disagrees with the patient, then, probably the next best agent that can be given is carbolic acid; from two to four drops may be administered in a teaspoonful of glycerine or olive oil, every four to six hours. I have no doubt that if we could apply carbolic acid directly to the ilium, it would arrest the ulcerative process immediately, and we would have but few if any more deaths from perforation of the intestines. It not only proves beneficial by its local effects, but when taken into the system, it acts as an antiseptic, and thereby counteracts the prostrating influence of the fever poison on the organic functions.

Owing to the idiosyncrasy, or whims of the patient, he cannot, or will not take our remedies; we must then substitute others in their place that may be more agreeable. I have found one or two grains of acetate of lead, and the sixth of a grain of morphine, given every four to six hours, to act promptly and with satisfactory results.

In this form of typhoid fever there is often a profuse and dangerous hemorrhage from the bowels, which, if not arrested speedily, may prove fatal in a few hours. An injection of half a drachm of Monsell's solution of persulphate of iron, in two ounces of water, is probably the most speedy remedy we have to check the morbid discharge. At the same time that we are using the enema, we may give the acetate of lead and opium by the mouth. If, after thirty-six hours, there has been no evacuation from the bowels, we must give an enema of half a pint of warm water, containing a table spoonful of salt. It will not be safe to give a cathartic, for that would start the bowels to running off again. In this form of the disease, where opium is so beneficial, and is required to be given so freely, the renal functions must be closely watched; and if the urine is scanty, some diuretic must be given at such intervals as to promote its flow.

*Treatment of Typho-Cerebral.*—The treatment of typhoid fever of the cerebral type must be conducted on the same general plan that has been already indicated. But our attention must be early directed to the delirium or head symptoms; for without sleep the patient will die, and that speedily. I have found nothing so beneficial in controling the delirium, and promoting sleep, as the following:—

| | | |
|---|---|---|
| ℞. | Chloroform, ........................................ | ʒij. |
| | Pulvis Acacia, } <br> Saccha Alba, } āā, ........................ | ʒiij. |
| | T. Opii Deodor, ........................................ | ʒij. |
| | Bromide Potassa, ........................................ | ʒiv. |
| | Aqua Mentha, ........................................ | ℥ij. |

Mix, and give a teaspoonful every two hours, until he is quiet.

The alterative and sedative must be given with the same restrictions as in the other cases. If the head is hot, it may be frequently bathed with cold water. If the bowels do not move regularly, the enema of warm water and salt may be used from time to time.

*Treatment of Typho-Gastric.*—The treatment of this type or complication requires a good deal of judgment, both as to the selection and use of remedies. Our first object must be to quiet the gastric irritation. To accomplish this, we give one grain of calomel, four grains of bicarbonate of soda, and one-sixth of a grain of morphine, every four hours, until the stomach is quiet. At the same time I give twenty grains of sulphite of soda, three or four times a day. I give this to arrest the fermentative process set up in the stomach by the typhoid poison.

If the above fails to correct the gastric disturbance in a reasonable time, we may combine two grains of acetate of lead, with the morphine, and leave out the calomel and bicarbonate of soda.

If the gastric irritation is not soon removed by the foregoing plan of treatment, or if the medicine is rejected by the stomach, then we may use the hypodermic injection, of the one-twelfth to the one-fourth of a grain of morphine; or we may blister the epigastric region, and sprinkle the denuded surface with morphine.

The diet should only consist of a few spoonfuls of milk porridge or ice cream. If the stomach is very irritable, it would probably be better not to give anything by the mouth except the medicines; and allow the patient to swallow small pieces of ice, if he is thirsty. It will be better to nourish the system by means of beef essence. One or two ounces may be thrown into the rectum, every four to six hours, and if it does not readily stay by the patient, a few drops of laudanum may be added to each dose.

A great deal of care is to be taken during convalescence; for there are probably more relapses in this variety than any of the others. Therefore, the patient should be restricted to a nutritious but unirritating diet, for a long time, until the digestive organs regain their tone.

*Treatment of Typhoid-Pneumonia.*—The treatment of this complication differs only from that of simple typhoid, in our efforts to overcome the pathological condition of the lungs. In order to meet this indication, we may give the following powders:—

℞. Hydrarg Chlor. Mite., ---------------- gr. v.
Pulvis Opii, ---------------------------- gr. v.
Sanguinaria, ---------------------------- gr. iij.

M. Fiant, chart vi. Give one every four hours, alternated with the following sedative cough mixture:—

℞. Syr. Scillæ Comp., ------------------------ ℥j.

Tinct. Sanguinaria, ------------------------ ℥j.

Camph. Tinct. Opii,------------------------- ℥j.

Tinct. Veratrum Viride, -------------------- ℥j.

Mix, and give a teaspoonful every four hours, until the fever abates, and the cough becomes loose. If the pulse is slow and feeble, we must leave out the veratrum, and continue the expectorant. The powders may be continued for twenty-four hours, or dispensed with sooner, if the tongue becomes moist. If the pain and breathing are troublesome, we may cup or leech the affected side, and cover the chest with raw onions, reduced to a pulp. This is by far the best external application that I have ever seen used in pneumonia—especially in children. I am not prepared to say what the physiological effects of onions are, or how they act therapeutically, but from my observation of their speedy action, I am inclined to believe that their oil contains anodyne, antispasmodic, and expectorant properties. For in a few hours after the application of raw onions to the chest, pain is often relieved; the quick and laborious breathing gives place to a quiet and refreshing sleep, and expectoration becomes easy.

If the patient is not better at the end of forty-eight hours, it may be well to apply a blister over the inflamed lung. Some have been deterred from the use of blisters, in this disease; but clinical experience has shown that there is but little trouble to be apprehended from their use, in this affection. I am, however, not a strong advocate for blisters; but, nevertheless I have seen some happy effects from their application.

If the fever assumes a low grade, with congestion of the capillaries, and a blueish tinge of the lips, we must then leave out the veratrum viride, continue the expectorant, and give the following as an organic stimulant, and to decarbonize the blood:—

| | | |
|---|---|---|
| ℞. | Potass Chloras, | ʒijss. |
| | Acacia, } Saccha Alba, } āā, | ʒiij. |
| | Aqua Mentha, | ℥iij. |
| | Chloroform, | ʒij. |

Mix, and give a teaspoonful every one, two, or four hours, owing to the urgency of the symptoms.

The chlorate of potash proves beneficial, by increasing the oxygen of the system, and thereby acts as an organic stimulant, arousing the organic functions to a healthy action. It is one of the best oxygenators of the blood that we have; and it should always be given in low forms of disease, where carbon is in excess in the system. Chloroform has a similar action, but it is more especially useful in quieting nervous irritation, allaying pain, and inducing sleep.

If the bowels should become moderately bound, and the tongue is dry and brown, two grains of calomel and five grains of Dover's powder may be given at bedtime, followed in the morning with one tablespoonful of castor oil, containing ten drops each of laudanum and spirits of turpentine. Purgation must be moderate, and the bowels closely watched, as in the other varieties.

If the fever be remittent, as is sometimes the case, two grains of quinine, and one grain each of opium and blood-root, given every four hours, will be the best treatment that can be adopted.

After the inflammation of the lungs has been arrested, and the patient begins to expectorate, we may discontinue the former expectorant, and give a tablespoonful, three or four times a day, of the infusion of half an ounce each of senega and asclepias tuberosa, to a pint of hot water.

The diet and tonics must be given during convalescence, as directed in simple typhoid.

*Treatment of Typho-Malarial Fever.*—Typhoid fever of a malarial type is sometimes a most difficult disease to treat, owing to the variable character of its symptoms. At one visit we find our patient sweating profusely, with symptoms pointing to a remittent fever; when we examine the pulse, we generally find it ranging from 120 to 140 per minute; and at our next visit we find all the indications of a continued fever. The diarrhœa, tympanitic bowels, dry skin, and hebetude of mind is an evidence that a typhoid poison is at work in the system. So the conflict seems to be between the morbific agent of typhoid fever on the one hand, and marsh malaria on the other, as to which shall gain complete possession of the system. For this reason, the case is somewhat troublesome to manage. If we treat it simply as a case of typhoid fever, we will certainly fail to cure the patient, or the disease will be greatly prolonged. For antiperiodics are to a certain extent demanded. And then, again, if we treat it as a case of remittent fever, with full doses of antiperiodics, it may be hazardous to our patient. We must, therefore, make a compromise, and select our treatment with some modification from that which is separately recommended for each disease.

As the gastric and portal systems are deranged, in this disease, our first object will be to correct them; and in order to accomplish this, we may give one grain of calomel, and four grains of Dover's powder, every four to six hours, until the tongue becomes moist, or gives evidence that the portal system is unlocked. If the stomach rejects the Dover's powder, we may substitute the one-sixth of a grain of morphine, for each dose.

After the secretions shall have been promoted, and the copious sweating comes on, we then discontinue the alterative, and give two grains of quinine, ten grains of chlorate of potash, and the eighth of a grain of morphine, every six hours, alternated with twenty grains of the sulphite of soda. Experience has demonstrated the fact that the latter is as efficacious in intermittent and remittent fevers, as it is in the aplastic diathesis. Therefore, the sulphites are eminently successful in typho-malarial fever.

Hiccough, which is so troublesome in this form of typhoid fever, is best treated with twenty drops of chloroform, and twenty grains of bromide of potash, rubbed up in a tablespoonful of syrup of acacia, and given at such intervals as to arrest the trouble.

The same percautions as to ventilation, diet, and the regulation of the bowels, must be observed in this, as in the other varieties. The diarrhœa must be checked by the turpentine emulsion; and the delirium can be combated with the chloroform mixture, as before mentioned. For a tonic and anti-peri odic, I have found the following very useful, *viz.*:—

℞. Tinct. Cinchonia, ........................ ℥j.<br>

Syr. Aurantii, ........................ ℥j.<br>

Salicine, ........................ ʒj.<br>

Arom. Sulph. Acid, ........................ ʒij.

Mix, and give a teaspoonful at each meal-time, and at bedtime.

In the treatment of all the grades of typhoid fever, our object should be to prevent or heal lesions, antidote the fever poison, remove the effete matter from the system, and promote the normal secretions; and then withdraw all medication except tonics, and husband the patient's strength by nutritious diet, ventilation, and cleanliness.

## Figures and Tables

**Figure f1:**